# The Effect of the Er^3+^:YAG Laser Decontamination Process on the Surface of Titanium Alloys, Depending on the Exposure Parameters

**DOI:** 10.3390/ma19040775

**Published:** 2026-02-16

**Authors:** Paulina Sobierajska, Maciej Dobrzynski, Kinga Grzech-Lesniak, Kinga Sekula, Damian Szymanski, Wojciech Zakrzewski, Krzysztof D. Dudek, Jacek Matys, Maria Szymonowicz, Katarzyna Wiglusz, Dawid Keszycki, Rafal J. Wiglusz

**Affiliations:** 1 Institute of Low Temperature and Structure Research, Polish Academy of Sciences, Okolna 2, 50-422 Wroclaw, Poland; d.szymanski@intibs.pl; 2 Department of Pediatric Dentistry and Preclinical Dentistry, Wroclaw Medical University, 50-425 Wroclaw, Poland; maciej.dobrzynski@umw.edu.pl; 3 Department of Integrated Dentistry, Faculty of Medicine and Dentistry, Wroclaw Medical University, Krakowska 26, 50-425 Wroclaw, Poland; kinga.grzech-lesniak@umw.edu.pl; 4 Department of Periodontics, School of Dentistry, Virginia Commonwealth University, Richmond, VA 23298-0566, USA; 5 Department of Advanced Manufacturing Technologies, Faculty of Mechanical Engineering, Wroclaw University of Science and Technology, Lukasiewicza 5, 50-371 Wroclaw, Poland; kinga.sekula@pwr.edu.pl (K.S.); dawid.keszycki@pwr.edu.pl (D.K.); 6 Pre-Clinical Research Centre, Wroclaw Medical University, Bujwida 44, 50-368 Wroclaw, Poland; wojciech.zakrzewski1992@gmail.com (W.Z.); maria.szymonowicz@umw.edu.pl (M.S.); 7 Department of Logistics and Transport Systems, Faculty of Mechanical Engineering, Wroclaw University of Science and Technology, 50-371 Wroclaw, Poland; krzysztof.dudek@pwr.edu.pl; 8 Department of Dental Surgery, Faculty of Medicine and Dentistry, Wroclaw Medical University, Krakowska 26, 50-425 Wroclaw, Poland; jacek.matys@umw.edu.pl; 9 Department of Basic Chemical Sciences, Faculty of Pharmacy, Wroclaw Medical University, Borowska 211 A, 50-566 Wroclaw, Poland; katarzyna.wiglusz@umw.edu.pl; 10 Meinig School of Biomedical Engineering, College of Engineering, Cornell University, Ithaca, NY 14853-1801, USA

**Keywords:** decontamination, Er^3+^:YAG, laser, peri-implantitis, Ti6Al4V, Ti6Al7Nb alloys

## Abstract

The dynamic development of laser therapy in dentistry is associated, among other factors, with the bactericidal effect of the energy emitted by laser devices. Therefore, they are also helpful for decontamination. They are increasingly used in the treatment of peri-implantitis, a bacterial inflammation of peri-implant tissues that is the most severe late complication of implantation and a potential cause of implant loss. Therefore, this study aimed to assess the safety of laser decontamination of the implant surface with respect to its effect on the integrity of the implant structure. In the present study, blocks of the titanium alloys Ti-6Al4V and Ti6Al7Nb were fabricated using electron-beam powder bed fusion and laser powder bed fusion, respectively. These alloys, commonly used in implantology, here in the form of Ti block scaffolds, have been exposed to an Er^3+^:YAG laser under various parameters (energy range of 50–320 mJ, exposure times of 20 or 30 s), and their effects have been further observed. To determine the changes induced by the laser, the following techniques were used: X-ray diffraction (XRD), Rietveld refinement method, scanning electron microscopy (SEM) with EDS (Energy-dispersive X-ray Spectroscopy), and thermography. The results show that the proposed Ti6Al4V and Ti6Al7Nb scaffolds can be exposed to an Er^3+^:YAG laser without damage when the power is limited to 0.5 W.

## 1. Introduction

Today, dental treatment with dental implants has irrevocably changed the field of dentistry. Although it has proven to be an effective procedure, with the increase in the use of implants, an emerging problem has become an increase in peri-implantitis [1,2,3]. Peri-implantitis is defined as the inflammation of soft and hard tissues around osseointegrated implants [4,5]. It eventually leads to periodontal pocket formation and loss of supporting bone. Among the reasons for peri-implantitis can be distinguished: poor oral hygiene, occlusion disorders, general diseases influencing healing processes, stress, genetic factors, and nicotine dependence [6]. Therefore, there is an urgent need for new solutions. A supplement or alternative to the above scheme may be the use of a laser to decontaminate the implant surface after cleaning from the deposits [7,8,9]. Lasers have wide applications in many areas of dentistry [10,11,12,13,14,15,16,17,18,19]. These devices emit light in the ultraviolet, infrared, or visible range through optical amplification via stimulated emission of electromagnetic radiation [20,21]. Er^3+^ ion-doped yttrium aluminum garnet (YAG) laser (Er^3+^:YAG), generating radiation at 2940 nm, has attracted significant interest for the removal of biofilm from dental implants [9,15,22,23]. However, the question of whether an effective treatment protocol is available without risking damage to the implant surface remains open.

Materials used in dentistry have several forms and must have specific properties for oral application [24,25,26]. Titanium alloys remain a dominant material in implantology. The mechanical properties and thus behavior of titanium implants depend on the 3D structure, pore volume fraction, and size distribution [27]. According to Adell et al. [28], almost all commercially available dental implants are made of titanium, and the pioneering work in this field is the Brånemark system, which offers high specific strength and biocompatibility [29]. Brånemark implants can form a thin oxide layer and thus possess outstanding corrosion resistance [26,30]. Within milliseconds of air exposure, an oxide layer forms on the exposed titanium surface [31]. This layer is retained at different pH values [32]. The success of implant fixation by osseointegration has been well established for many reconstructive procedures [33,34,35,36]. Osseointegration is a process occurring after successful implantation. It can be described as a foreign body reaction leading to the formation of interfacial bone during a defense reaction to shield off the implant from the tissues [37]. According to recent scientific works, it is a physiological immune-driven process [38,39].

Dental implants, although placed in an unsterile environment, maintain a relatively low infection rate of approximately 1% [40]. After the healing period, bacteria may be absent from the bone-anchored portion of the implant, and, after establishing a proper bacterial seal, from the portion of the implant that penetrates soft tissues. It prevents solid medical implants from being recognized by the host tissue as its own. Inflammatory immune balance can be altered by factors such as implant location, material chemistry, mechanical stress, or surgery [41]. According to Albrektsson T. et al. [42], successful implantations are a delicate balance of a positive immune response tailored to the individual host’s immune system. Titanium implants activate various immunological components in bone, such as macrophages, complement, and neutrophils. Titanium activates the immune system early and inhibits bone resorption. Eventually, it leads to the isolation of the titanium implant due to the formation of a surface sheath of bone during osseointegration. It is essential to recognize that it can be disrupted by external factors and, ultimately, lead to implant failure due to immune and inflammatory processes [43].

Ti6Al4V and Ti6Al7Nb are two of the most commonly used implantation alloys [44,45] that overcome the poor mechanical strength of pure titanium. Both alloys have mechanical properties similar to those of stainless steel and Co-Cr alloy [46]. However, due to the toxic effects of releasing aluminum (Al) and vanadium (V) into the human body, Ti6Al7Nb is a better candidate for oral surgery. It is characterized by high corrosion resistance and significant biocompatibility [47,48].

In recent years, the understanding of peri-implantitis and the optimization of implant decontamination protocols have evolved substantially. Recent studies highlight the growing importance of minimally invasive decontamination techniques, including Er^3+^:YAG laser irradiation [49] and emphasize the need to balance antimicrobial efficacy with preservation of implant surface integrity. Therefore, updating existing knowledge with recent literature is essential to position the present study within current scientific and clinical developments.

For the first time, this paper demonstrates the safe use of an Er^3+^:YAG laser on titanium implant surfaces (Ti6Al4V and Ti6Al7Nb) to maintain proper oral hygiene. The primary objective was to determine whether peri-implantitis treatment could be applied to the implant surface to eliminate soft and hard deposits while avoiding damage to the alloy topography. XRD, SEM, EDS, and thermography were used to characterize the two types of Ti-cubic blocks fabricated and to detect any alteration of the implant surface after laser irradiation under various working parameters. Destruction of the surface may induce changes in the oxide layer, which impairs osseointegration of the implant [50,51,52]. These two distinct pore configurations of the titanium implant were intentionally selected to assess how pore geometry and dimensions influence cell attachment, tissue ingrowth, and vascularization potential. Smaller pores (~350–400 µm) generally support osteoblast adhesion and early bone matrix formation, whereas larger interconnected pores (~1–1.5 mm) enhance nutrient diffusion and neovascularization. Additionally, the distinct pore architectures were designed to evaluate how the scaffolds perform under laser-based processing and treatment conditions, as laser energy absorption, distribution, and thermal effects may vary depending on pore size and geometry. Comparing the two configurations, therefore, allows us to determine which design is more suitable for laser-assisted applications.

## 2. Materials and Methods

### 2.1. Scaffold Fabrication

Samples in the form of open porous structures were designed and fabricated as described in another paper [53].

**Figure 1:**
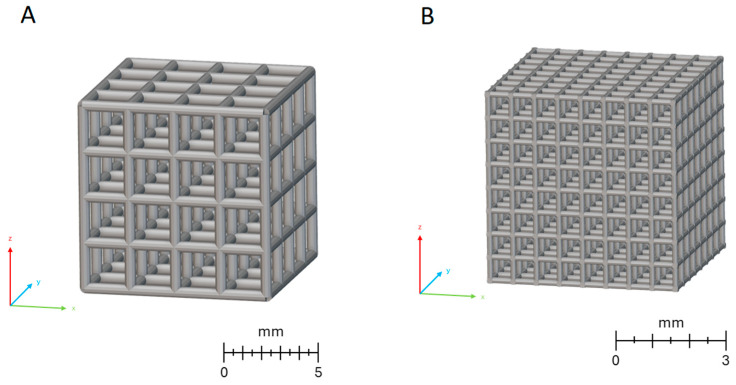
3D models of (**A**) Ti6Al4V and (**B**) Ti6Al7Nb scaffolds.

A model of a porous unit cell composed of struts was designed in a cubic form using computer-aided design (CAD) and Magics software (15.0, Materialise HQ, Leuven, Belgium). The tested samples, in the shape of a 10 × 10 mm cube (Figure 1A), were produced using standard Ti6Al4V Arcam powder with particle sizes ranging from 40 to 106 µm, and an electron-beam powder bed fusion (PBF-E/M) device (EOS, Arcam A1, Frösön, Sweden). The chemical composition (wt.%) of the Ti6Al4V powder complied with the ASTM F1472–14 standard.

Additionally, a smaller cubic scaffold (Figure 1B) with external dimensions of 4.95 × 4.95 × 4.95 mm was tested. It was fabricated using a laser powder bed fusion of metals (PBF-L/M) system (ReaLizer 50, Borchen, Germany) with Ti6Al7Nb alloy powder (TLS Technik GmbH & Co Spezialpulver KG, Bitterfeld-Wolfen, Germany), composed of spherical particles with diameters ranging from 20 to 63 µm. According to the manufacturer’s certificate, the chemical composition (wt.%) of the Ti6Al7Nb powder was: 5.82% Al, 6.58% Nb, 0.050% Fe, 0.165% O, and Ti as the remainder. The contents of the determined elements complied with the requirements of ISO 5832-11 [54].

### 2.2. Material Characterization

The structure and morphology of raw Ti6Al7Nb (as presented by Wiglusz et al. [55]) and Ti6Al4V powders were studied using X-ray diffraction (XRD) technique and scanning electron microscopy (SEM). The XRD patterns were measured in the 20–60° 2*θ* range by using an X’Pert Pro PANalytical X-ray diffractometer (Malvern Panalytical Ltd., Royston, UK) equipped with Ni-filtered Cu Kα_1_ radiation (Kα_1_—1.54060 Å, V—40 kV, I—30 mA). The experimental XRD patterns were compared with the standard from the Inorganic Crystal Structure Database (ICSD nos. 191187 and 38755 [56,57]) and analyzed. The Rietveld refinement of the XRD patterns (Figure S1) was carried out based on the hexagonal crystal structure of titanium and the cubic structure of titanium oxide, with better approximation and indexing of the Crystallographic Information File (CIF) using the Maud program (version 2.999) [58,59].

The morphology and microstructure of titanium alloys and titanium scaffolds were examined by using a Field Emission Scanning Electron Microscope (FE-SEM) (FEI Nova NanoSEM 230, Hillsboro, OR, USA) equipped with an energy dispersive X-ray spectrometer (EDAX Genesis XM4, Hillsboro, OR, USA) with a resolution better than 135 eV and compatible with Genesis EDAX microanalysis Software (version 6.0). The Ti6Al7Nb and Ti6Al4V powders and scaffolds were analyzed by electron beam at low accelerating voltage at 5.0 kV using a secondary electron (SE) detector (Hillsboro, OR, USA). The SEM images were recorded in low-accelerating-voltage mode to show detailed morphological features of the examined materials. EDS spectra were obtained from the flat surfaces of the samples at 30.0 kV. X-ray signals were collected from five selected areas to ensure satisfactory statistical averaging. The grain size distribution has been determined with the help of the freeware software ImageJ 1.53k [60], which measures the particle diagonal lines in the images. For about 200 particles, the particle diameters have been calculated as the median value of the two diagonal lines. The SurfCharJ plugin for ImageJ was used to estimate surface roughness parameters: mean roughness (*R_a_*) and root-mean-square roughness (*R_q_*). The 3D image tool Surface Plot was used to visualize the surface topography.

### 2.3. Laser Irradiation

The samples were irradiated with a 2940-nm Er^3+^:YAG laser (LightWalker, Fotona, Ljubljana, Slovenia) using a tipless handpiece (H_02_, Fotona). Individual samples were treated with various laser parameters to investigate their effects on the material’s surface. Various parameters were tested according to Variant I: energy 80 mJ, power 0.8 W, frequency 10 Hz, spot 0.9 mm, pulse width 100 µs, cooling 4 air; 6 water (where 4 indicates the airflow intensity, and *6* indicates the water spray intensity. These numerical settings correspond to a water flow of approximately 30 mL/min in the LightWalker system and define the cooling applied during irradiation); Variant II: energy 160 mJ, power 1.6 W, frequency 10 Hz, spot 0.9 mm, pulse width 100 µs, cooling 4 air; 6 water; Variant III: energy 320 mJ, power 9.6 W, frequency 30 Hz, spot 0.9 mm, pulse width 100 µs, cooling 4 air; 6 water. Additionally, Variant IV with the lowest laser power was tested: energy 50 mJ, power 0.5 W, frequency 10 Hz, spot 0.9 mm, pulse width 100 µs, cooling 4 air; 6 water. Laser radiation was emitted under the control of a ThermaCAM P640 thermal imager (FLIR) with a visible range of 7.5–13 μm. Changes in the surface temperature distribution of the samples during cooling were recorded at 5 Hz. Exposition time was either 20 s or 30 s, and samples were divided into several types visible in Figure S2. All temperature measurements were conducted at a room temperature of 20.5 °C (*T*_0_).

The thermogram sequence was analyzed using ThermaCAM Researcher Pro. For statistical analysis of measurement results, the STATISTICA v.13 program (StatSoft, Inc., Tulsa, OK, USA) was used. Since the surface temperature distributions of the samples deviated from normality (as verified by the Shapiro–Wilk test), nonparametric tests were used in the statistical analysis. The significance of the maximum temperature differences was verified using the Mann–Whitney U test for two samples, the Kruskal–Wallis test for three samples, and Dunn’s test as a post hoc test. In all two-sided statistical tests, a *p*-value < 0.05 was considered significant.

## 3. Results

### 3.1. Titanium Alloys and Scaffolds Characterization

**Figure 2:**
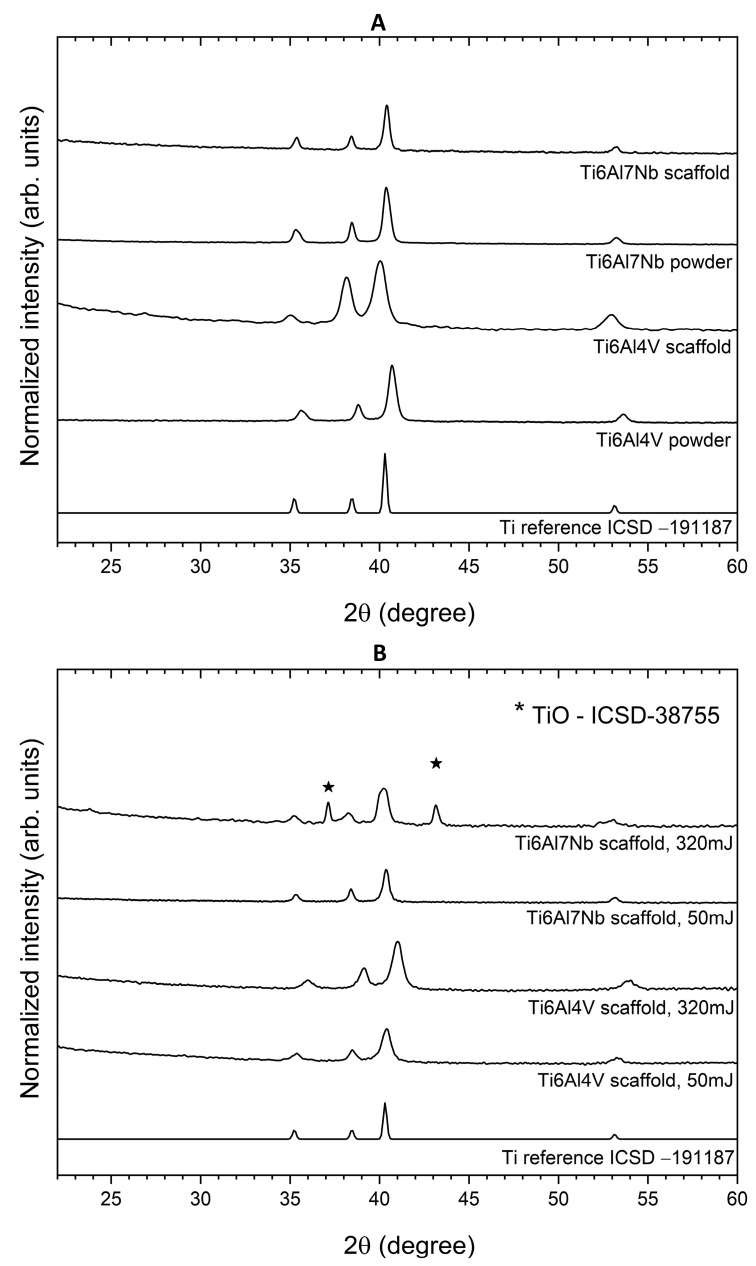
X-ray powder diffraction patterns of the Ti6Al4V and Ti6Al7Nb powders (**A**) and scaffolds before and after laser exposure, 50 mJ and 320 mJ (**B**). Symbol * identifies the TiO-ICSD-38755 phase.

The crystal structures of the Ti6Al7Nb [61] and Ti6Al4V alloy-powders were observed by the XRD measurement (see Figure 2A). The pure hexagonal phases corresponding to the reference standard (diffraction peak positions) of Titanium (ICSD–191187) [57] were identified in all samples. However, the XRD patterns of the scaffolds differ from those of the powders, as the scaffolds exhibit broader, slightly shifted peaks, indicating smaller crystal size, larger microstrains, or residual stresses. Both materials mainly contain the α-Ti (hcp) phase, characterized by reflections at (100), (002), and (101) in the 35–40° (2*θ*) range. However, a trace amount of the β-Ti (bcc, cubic) phase may also be present, as evidenced by the (110) peak near 38° and additional reflections, such as (200) at ~55°. However, they are difficult to distinguish at such low levels, and therefore, the α-alpha phase may broaden. These differences arise from local melting and rapid solidification during additive manufacturing, which can partially transform the α-phase into the β-phase and introduce thermal stresses. Moreover, the Rietveld refinement was performed to determine the unit-cell parameters of all studied materials. The formation of the hexagonal phase of titanium alloys and TiO was confirmed. Details are displayed in Table 1 and Figure S1. As shown, no trend in cell parameters or cell volume is observed.

To assess the effect of laser exposure on the structure of both Ti cubic scaffolds, XRD was performed at laser powers of 50 mJ and 320 mJ (Figure 2B). As shown in Figure 2, for the Ti-V scaffold and both energy levels, no additional phases form; only a shift in peak positions is observed. In the case of Ti-Nb scaffolds, we can see that 50 mJ does not affect the scaffold structure (no peak shifts, no additional peaks, and no significant changes in FWHM). However, at a laser energy of 320 mJ, we observe changes in the diffractogram, broadening of the peaks, and the appearance of an additional phase, identified as cubic TiO (titanium(II) oxide, ICSD–38755, space group: *Fm*$\bar{3}$*m* (225)) in 26.9% to the 73.1% of the Ti6Al7Nb scaffold (Table 1). This effect will be discussed in more detail based on the SEM-EDS results. Additionally, it can be observed that there are larger shifts in the peaks for Ti-V than for Ti-Nb with increasing laser energy, toward higher values of 2θ for Ti-V and toward lower values of 2θ (more subtly) for Ti-Nb. The observed peak shifts indicate changes in the crystal lattice parameters caused by the laser. In the case of the Ti-V scaffold, the peaks shift towards higher 2θ values, suggesting a decrease in the interplanar distance (*d* parameter) in accordance with Bragg’s law. This may be due to compressive stresses or slight shrinkage of the crystal lattice caused by laser treatment. In the case of the Ti-Nb scaffold, however, the peaks shift slightly towards lower 2θ values, which means an increase in the d parameter, which may result from residual tensile stresses or local structural relaxation. The difference between Ti-V and Ti-Nb is likely attributable to differences in alloy composition and microstructure, which affect the materials’ responses to thermal gradients and rapid cooling during laser exposure. Also, the laser beam scatters differently in smaller pores (Ti-Nb) than in larger ones (Ti-V).

**Figure 3:**
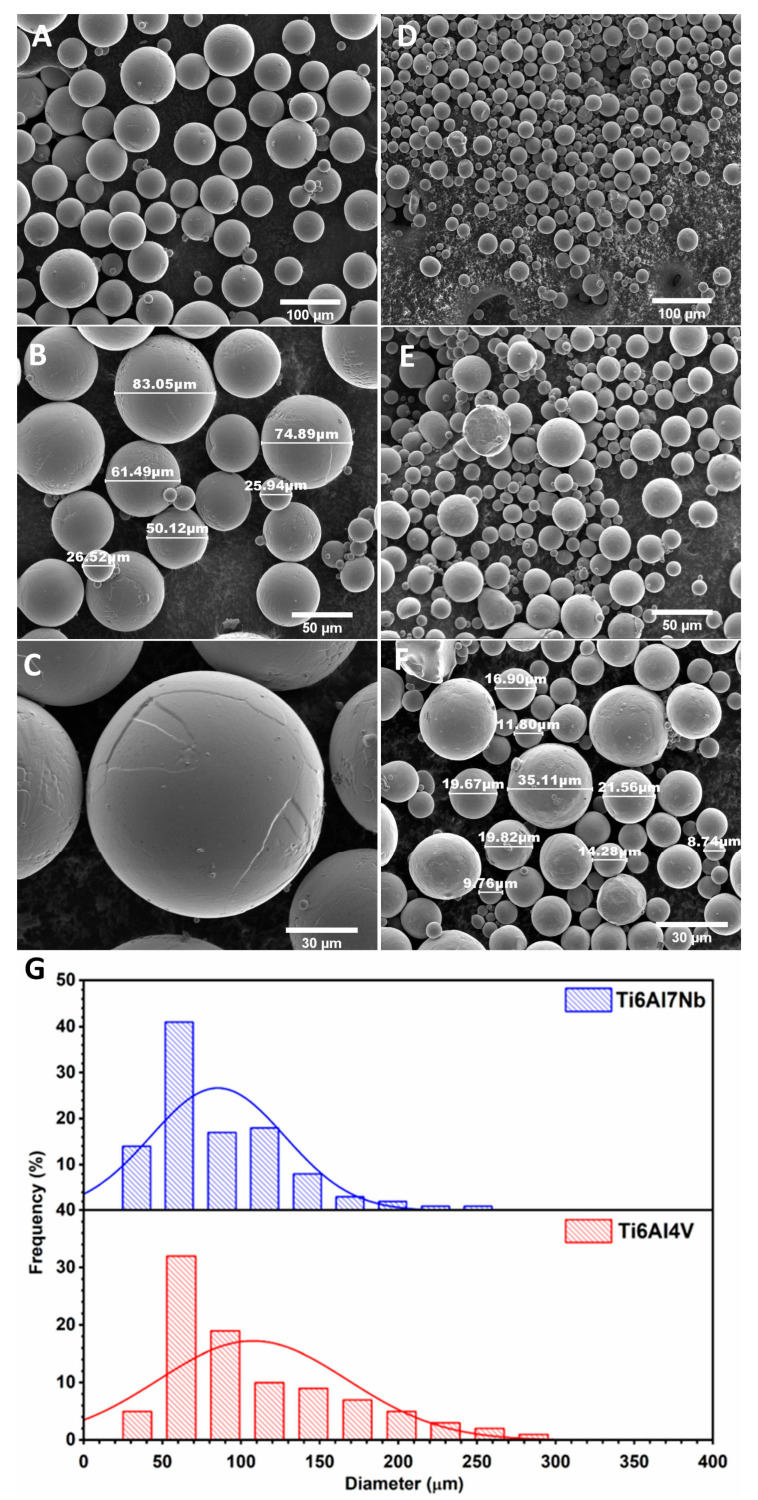
SEM images of the Ti6Al7V (**A**–**C**) and Ti6Al4Nb (**D**–**F**) powders with the indication of topography, spherical morphology, and size of the particles. Histograms of the particle size (**G**).

To determine the particle size and morphology of the Ti6Al7Nb and Ti6Al4V powders, SEM measurements were performed. Figure 3A–F shows representative SEM images of the examined titanium alloys, which reveal that Ti6Al7Nb and Ti6Al4V particles are micronized and exhibit similar morphologies. For both samples, a regular morphology with particle diameters ranging from 40 to 300 µm was observed. The size-distribution histograms (Figure 3G) indicate that the maximum particle size increased from 90 µm to 110 µm for Ti6Al7Nb and Ti6Al4V, respectively. It can also be seen that spherical particles are non-aggregated and uniformly distributed. A more detailed SEM analysis at high magnification also showed that the particle surfaces of both materials exhibit defects (cracks) and irregularities.

**Figure 4:**
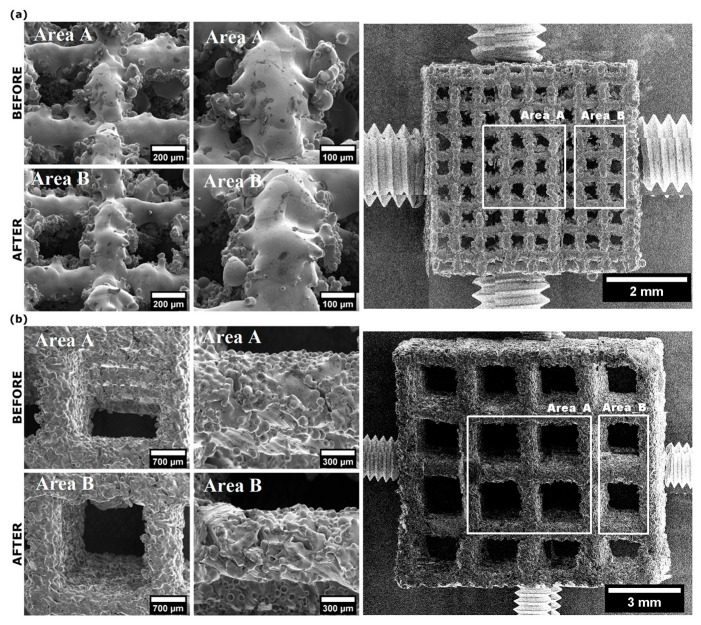
SEM images of Ti6Al7Nb (**a**) and Ti6Al4V scaffold samples (**b**) before and after 50 mJ, 0.5W, 10 Hz, 0.9 mm, 100 µs, cooling 4 air; 6 water, 30 s of laser exposure.

The SEM images of the manufactured Ti6Al7Nb and Ti6Al4V scaffolds before and after laser exposure are presented in Figure 4. The laser power and exposure time were 0.5 W (50 mJ) and 30 s, respectively. As shown in Figure 4a, the Ti6Al7Nb scaffold exhibits a relatively smooth surface and a well-organized pore structure, with pores completely interconnected before and after laser exposure. As shown in the magnified SEM images (see Figure 4a, right side), the observed structures of the Ti6Al7Nb scaffold are free of surface defects and cracks. It appears that laser exposure for 30 s at 0.5 W does not alter the morphology of the Ti6Al7Nb scaffold.

The differences between the morphologies of Ti6Al4V and Ti6Al7Nb scaffolds are clearly visible, as shown in Figure 4b. SEM images of the Ti6Al4V scaffold show a well-defined, micro-sized pore structure with a rough surface. On the other hand, the surface of the Ti6Al7Nb scaffold is more irregular, with unmelted and partially melted metal particles visible (Figure 4a). Moreover, the SEM images of the Ti6Al4V recorded at higher magnification showed that globular-shaped deposits could be distinguished in the areas between the pores. However, a more detailed SEM analysis revealed that the images of the Ti6Al4V scaffold recorded before and after laser exposure are very similar, indicating no effect on laser-induced morphological changes.

To determine the limiting values of laser power and exposure time, the examined materials were irradiated with a laser at power levels from 0.8 to 3.2 W for 20 and 30 s. As shown in Figure S3, for the Ti6Al7Nb scaffold, all applied laser powers for 20 s resulted in numerous surface defects and cut edges. Moreover, across all applied laser powers, both deep holes and thinner gaps were observed in the Ti6Al7Nb scaffold. The same results are observed for a 30 s laser exposure at 0.8, 1.6, and 3.2 W (see Figure S4). For the Ti6Al4V scaffolds, similar results were observed with the same laser exposure parameter (Figures S5 and S6).

It is worth emphasizing that the pore size of both cubic Ti-based scaffolds could be estimated from the SEM images (Figures S3–S6). For the Ti6Al7Nb block, the pores have a size in the range of 350–415 µm, while for the Ti6Al7V block, the pores have a diameter in the range of 1.4–1.6 mm.

Studies using XRD and SEM techniques showed that implant morphology did not change in either titanium alloy, Ti6Al7Nb or Ti6Al4V, when a low-power Er^3+^:YAG laser was used. High levels of Er^3+^:YAG laser use have led to damage in the implant surface, which is a considerable risk factor for periimplantitis.

Moreover, experimental evidence shows that high laser power, regardless of exposure time, promotes the formation of oxide phases. As shown in Table 2, EDS data indicate that the oxygen (O) content in the Ti6Al7Nb alloy increases with increasing applied laser power. This finding aligns with the conclusions drawn from XRD studies, which have confirmed the formation of TiO (titanium(II) oxide) on the surface of the material under investigation (see Figure 2). It is also noteworthy that the confirmed EDS tests did not reveal the formation of any additional impurities, such as carbon. For the Ti6Al4V alloy, no correlation was observed between the formation of oxide phases and laser power (see Table S1). This phenomenon can be attributed to the inherent limitations of the EDS method. Quantitative determination of oxygen (O) and vanadium (V) in inorganic compounds by EDS is subject to significant uncertainty due to inherent limitations of the technique. Quantitative determination of oxygen (O) in vanadium (V) compounds by EDS is complicated by the overlap of the O-*Kα* line with low-energy vanadium L lines, hindering reliable peak deconvolution. Quantification is further affected by matrix correction errors (ZAF/φ(ρz)), which are particularly pronounced for light elements in the presence of heavier elements such as vanadium.

**Figure 5:**
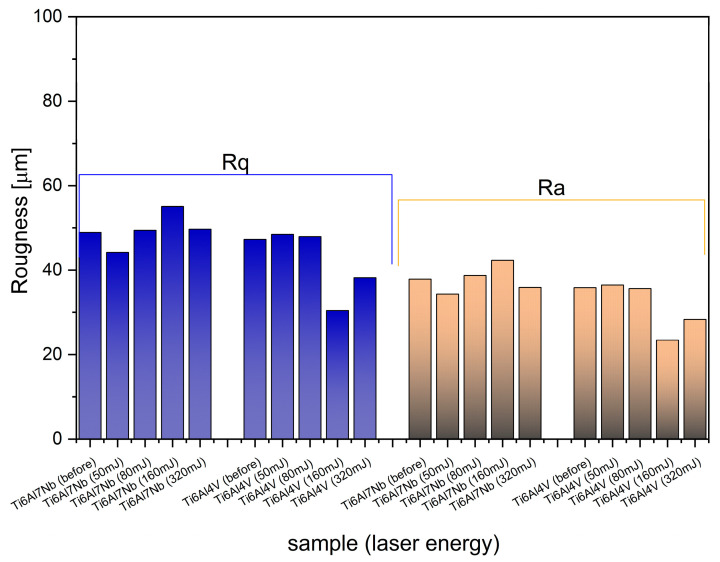
Surface roughness parameters: average roughness (*R_a_*) and root mean square (*R_q_*) for Ti cubic scaffolds (Ti-V and Ti-Nb) before and after exposition on different laser energies (30 s).

The roughness parameters for the Ti scaffold surface were estimated from SEM images using ImageJ and SurfCharJ plugin [62]. The results presented in Figure 5 show that *Rq* (root mean square roughness) is higher than *Ra* (average roughness), which is as expected, since *Rq* is more sensitive to large deviations, such as sharp peaks and deep valleys, while *Ra* represents the arithmetic mean of height deviations and is less susceptible to the influence of extreme values. Therefore, a higher *Rq* indicates pronounced surface irregularities, confirming a high texture. In this case, both parameters are approximately 40 µm (*Ra*) and 50 µm (*Rq*) for 50 mJ of laser energy, reflecting the very rough surface typical of porous scaffolds. Such high roughness has obvious consequences: it increases the surface area and topographical complexity, which can improve cell adhesion, proliferation, and osseointegration. However, excessive roughness can also lead to stress concentrations, reduced mechanical strength, and potential difficulties with cleaning or sterilization.

**Figure 6:**
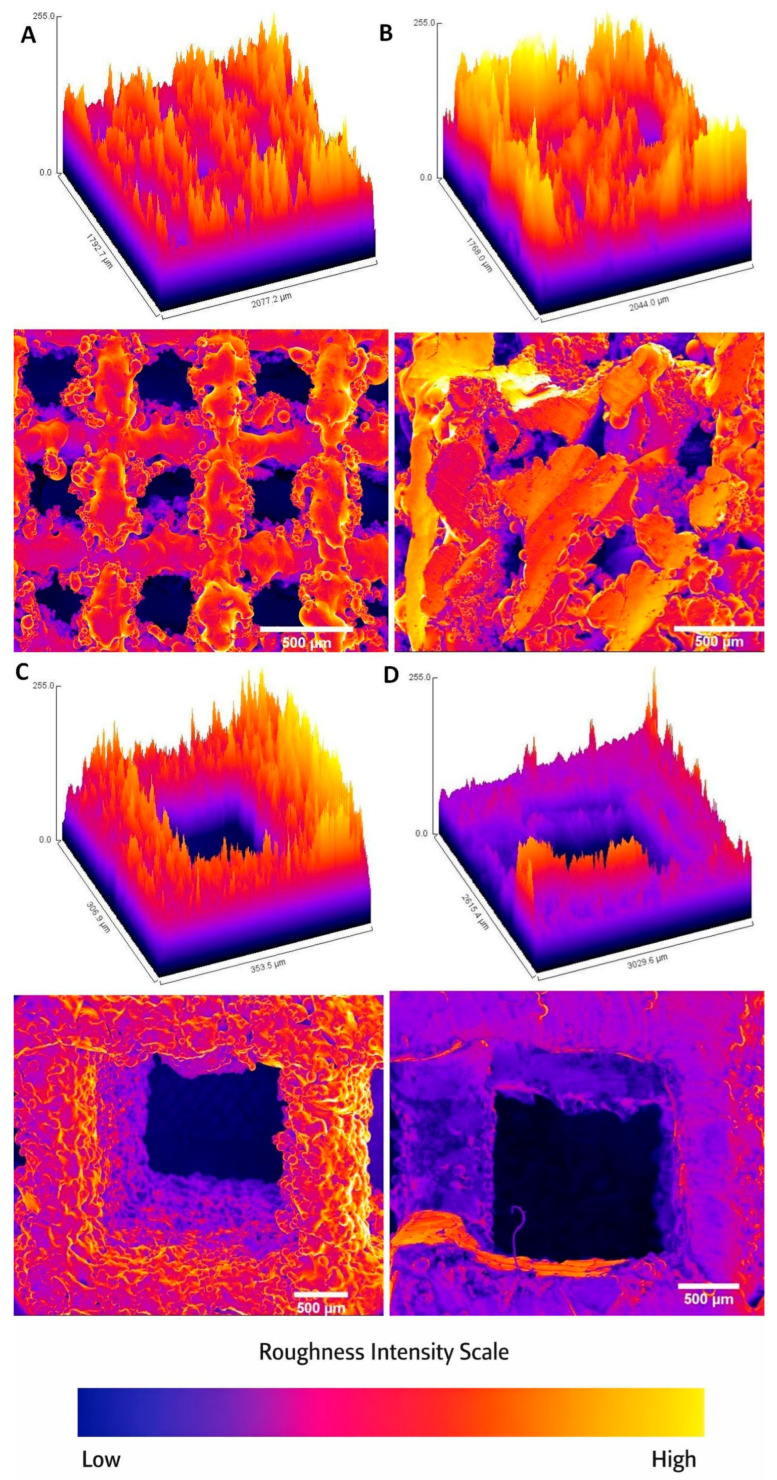
3D reproduction (Surface Plot) of (**A**) Ti-Nb, 50 mJ, (**B**) Ti-Nb, 160 mJ, (**C**) Ti-V, 50 mJ, (**D**) Ti-V, 160 mJ. The color scale runs from dark blue/purple (lowest elevations) through magenta and red to orange and finally light yellow (highest peaks), with each color representing increasing elevation values according to the roughness intensity scale.

The effect of laser energy on roughness varies with scaffold type. Figure 6 presents a 3D reproduction (Surface Plot) of some variants of the tested alloys under different energies of 50 mJ and 160 mJ, respectively. For Ti-V, *Ra*, and *Rq* decrease with increasing laser energy, reaching their lowest values at 160 mJ, with an overall change in *Rq* of approximately 20 µm, suggesting significant surface smoothing or partial melting. In contrast, for Ti-Nb, roughness increases with laser energy up to 160 mJ, with a maximum change in *Rq* of 10 µm, indicating roughening. These trends reflect various alloy compositions and microstructures that influence the thermal response to rapid heating and cooling.

At a laser power of 50 mJ, roughness values are approximately 44 µm for Ti-Nb and 48 µm for Ti-V. The 4 µm difference is slight (~8%) and unlikely to significantly affect biological performance. Also, the difference between 50 mJ and before laser irradiation is not significant in both cases of the Ti scaffolds. Instead, when selecting materials for dental implants and applying laser decontamination, biocompatibility and surface stability under laser irradiation should be prioritized. Ti-Nb stands out as the preferred option: niobium is non-toxic and corrosion-resistant, whereas vanadium released from Ti-6Al-4V alloys can exhibit cytotoxic effects when it corrodes or wears off.

### 3.2. Thermography Evaluation

**Figure 7:**
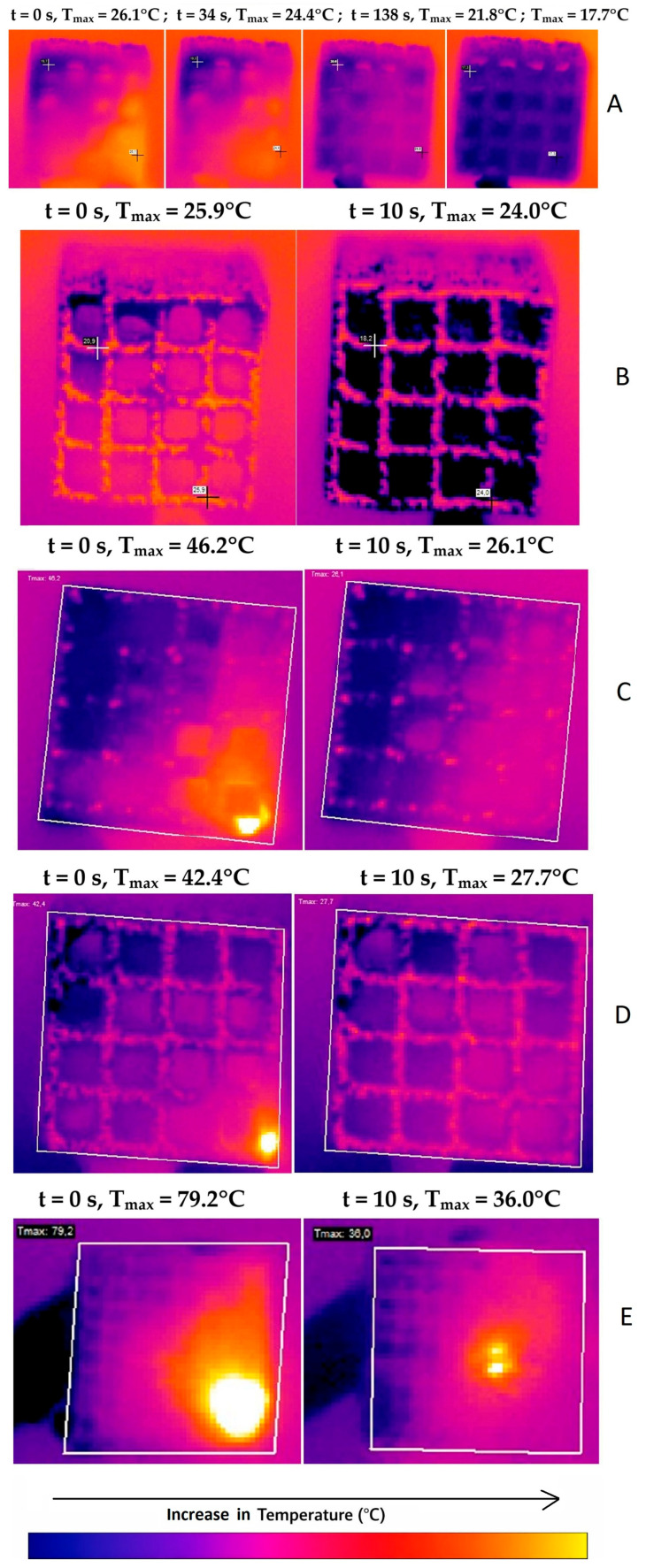
Thermograms of the Ti-V sample heated by laser to (**A**) 26.1 °C, (**B**) 25.9 °C, (**C**) 46.2 °C, (**D**) 42.4 °C during water and air cooling. Thermograms of the Ti-Nb sample (**E**): maximum temperature without water cooling (**left**) and with water.

The sequences of each test (Table 3) were recorded as thermograms and analyzed to obtain the location and the surface temperature function over the entire validity period. Figure 7 shows examples of thermograms illustrating the temperature distribution on selected combined active surfaces after accounting for laser heating (*t* = 0 s), along with several time points.

The Mann–Whitney and Kruskal–Wallis tests were used to evaluate whether the maximum temperatures differed significantly between independent groups. The *p*-values reported in the figures (all >0.05) indicate that the differences among the groups compared (material type, surface finish, and exposure time) were not statistically significant. The *Z*-values reflect the magnitude and direction of the rank differences. In our results, their low absolute values confirm that the temperature distributions between these groups were highly similar. Thus, these tests demonstrate that these factors did not meaningfully influence the maximum temperature during laser irradiation.

**Figure 8:**
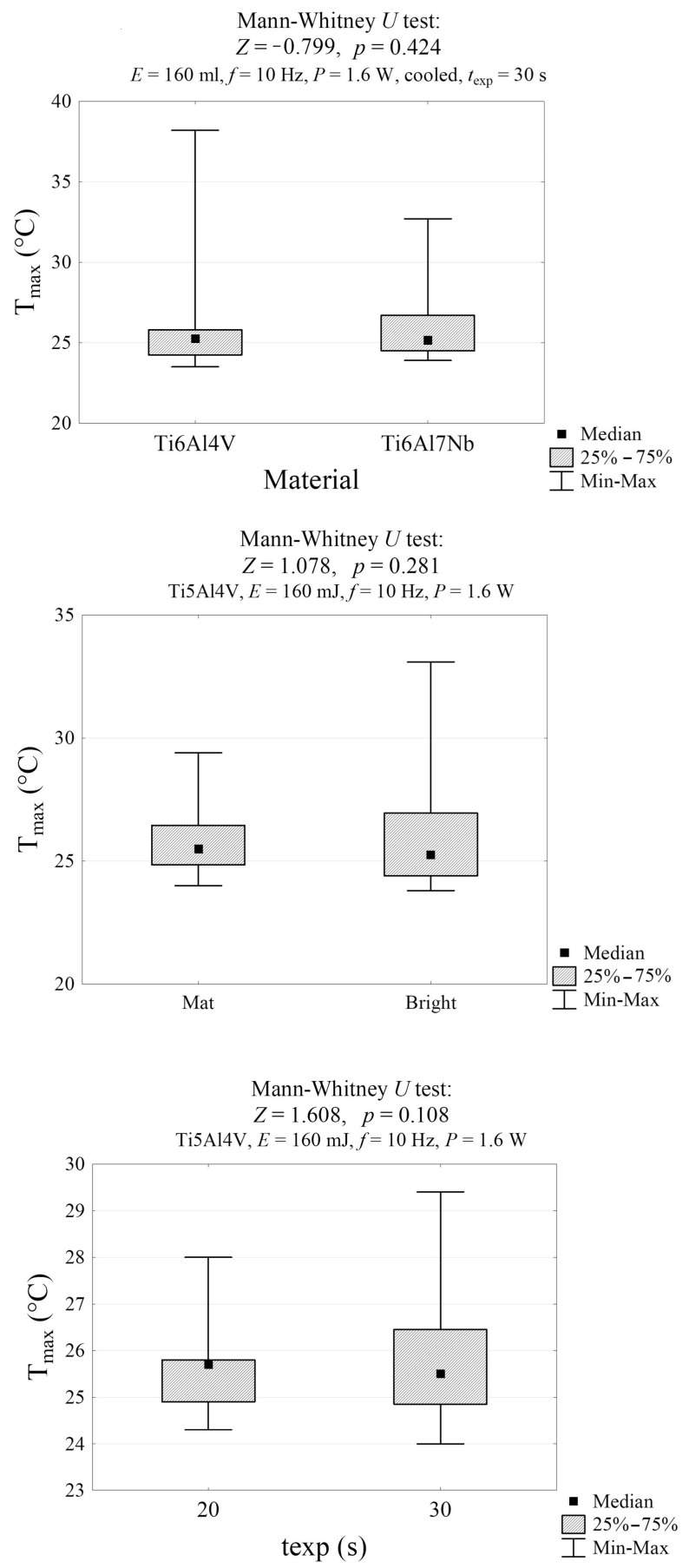
Maximum temperature of the sample during laser exposure in groups differing in material, color, and exposure time, along with the results of significance tests. The emissivity factor of the bright surface is *ε_b_* = 0.27, and the emissivity factor of the matte surface is *ε_m_* = 0.75.e.

**Figure 9:**
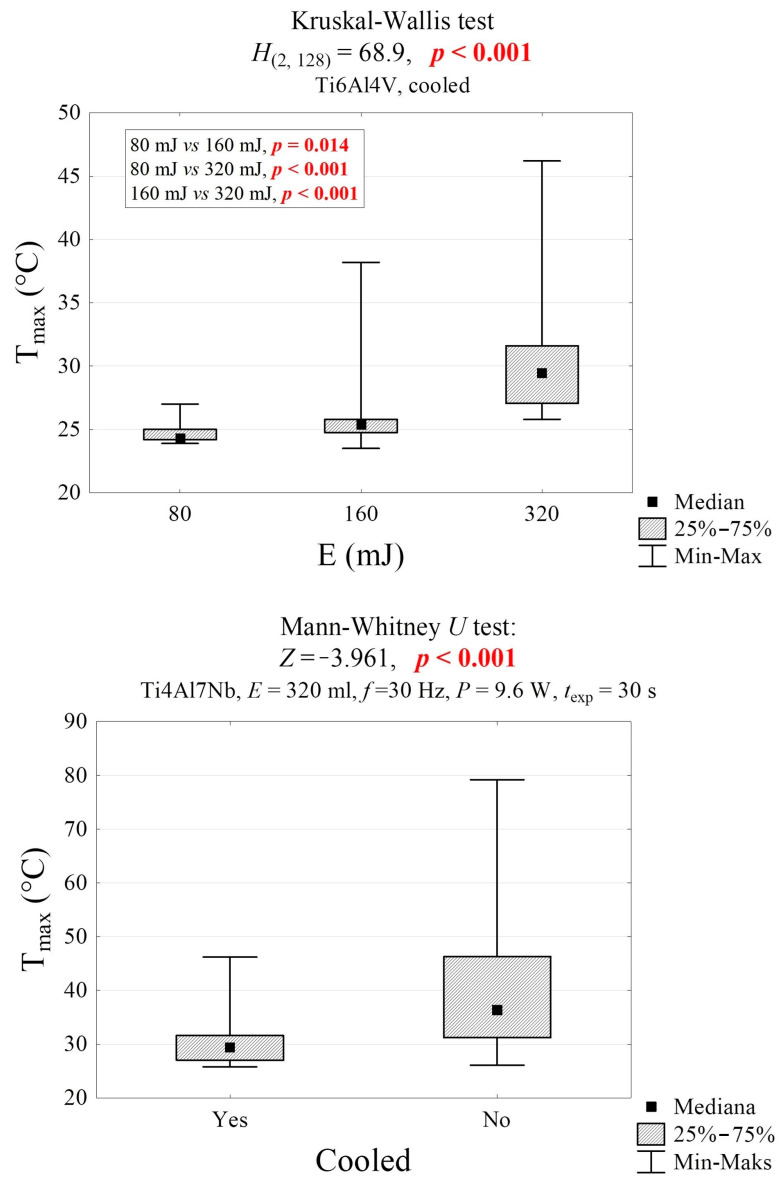
Maximum temperature of the sample during laser exposure in groups differing in laser energy and water cooling, along with the results of significance tests.

**Figure 10:**
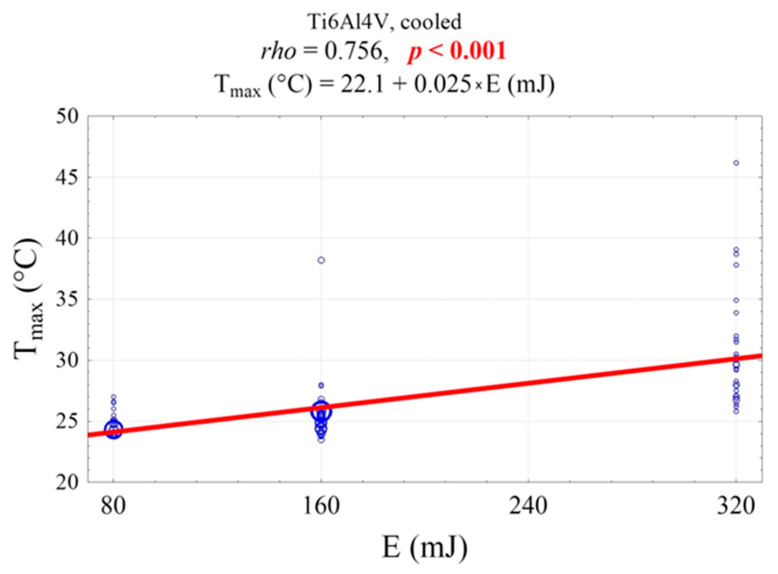
Scatter plot of the sample’s maximum temperature as a function of laser energy, including the Spearman rank correlation coefficient (rho) and the linear regression equation. All data points (blue dots) represent the average of three repeated measurements, and the same cooling conditions were applied for each laser energy level.

The sample’s maximum temperature was not significantly affected by the material, color, or exposure time (Figure 8). However, it was significantly influenced by the laser energy and the cooling conditions (Figure 9 and Figure 10). The temperature of the air-cooled Ti6Al4Vb sample exposed to laser radiation with an energy of 80 mJ is significantly lower than that of the sample exposed to the laser with an energy of 160 mJ (24.3 °C vs. 25.5 °C, *p* = 0.014) and the laser with an energy of 320 mJ (24.3 °C vs. 29.5 °C, *p* < 0.001). Furthermore, the temperature of the sample exposed to the laser with an energy of 160 mJ is significantly lower than that of the sample exposed to the laser with an energy of 320 mJ (25.5 °C vs. 29.5 °C, *p* < 0.001). The surface temperature of the water-cooled Ti6Al7Nb sample is lower than that of the non-cooled sample by an average of 11.7 °C (25.2 °C vs. 36.4 °C, *p* < 0.001, Figure 9).

**Figure 11:**
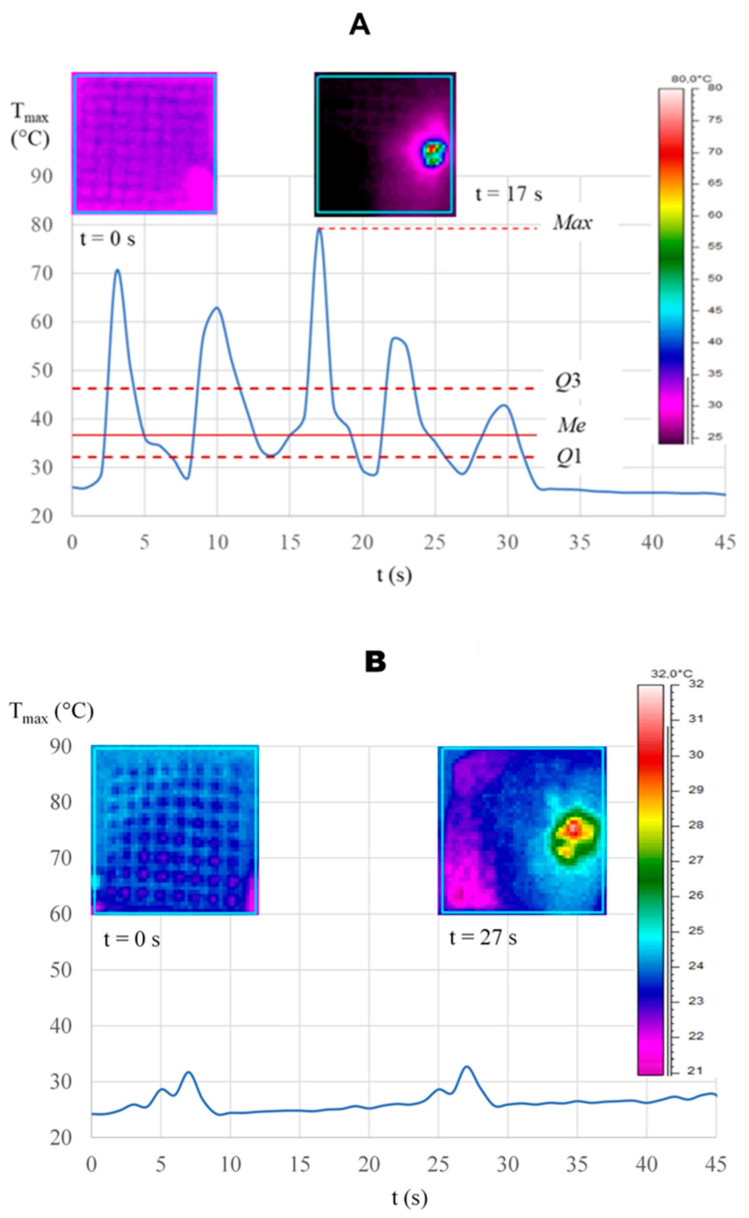
Profile of the maximum temperature of the Ti6Al7Nb sample during laser exposure at *P* = 1.6 W, *f* = 30 Hz, and *E* = 320 mJ, without (**A**) and with (**B**) water cooling.

A significant positive correlation was observed between the sample’s maximum temperature and laser energy (Figure 11). An increase of 1 mJ in energy was associated with an average increase in maximum temperature of 0.025 °C (Figure 10). The temperature fluctuations observed in Figure 11 are related to the scaffold’s porosity. During the 30-s irradiation period, the operator’s hand movements intermittently caused the laser beam to contact both solid metal areas and porous regions. This natural variation in surface characteristics caused the temperature to rise and fall, even though the laser beam itself was neither on nor off.

## 4. Discussion

An Er^3+^:YAG laser can be used successfully for ablation of target tissues and can reduce bacterial contamination due to its unidirectional light and side-firing tips. The laser beam allows access to all irregularities of the implant surface [63]. It is important to note that Er^3+^:YAG has to be used at a limited power during the ablation process [64]. When the pulse energy and irradiation time increased, greater surface alterations, including surface flattening and microfractures, were observed [23,55,65]. The observations under the SEM microscope showed no changes in the material’s surface after decontamination of the tested titanium alloys with the Er^3+^:YAG laser at low power (0.5 W). It has been shown that high power (9.6 W) can damage the alloy surface, which, in the case of implants, is a disadvantage in the treatment of peri-implantitis, as it creates microscopic irregularities that are susceptible to bacterial colonization. The temperature measurements during laser operation were recorded. Ti6Al7Nb samples exhibit porous structures that are promising for replacing damaged bone tissue; their mechanical properties are similar to those of bone, and integration with tissues is facilitated by material porosity [55]. In turn, the Ti6Al4V alloy is used in orthopedic surgery and dentistry as an implant material, enabling the reconstruction of missing teeth [1]. Medium-Strength Surgical Implant Alloy (Ti6Al7Nb) was produced, especially for the assembly of femoral surgical elements for hip prostheses. Its metallurgy is nearly comparable to that of Ti6Al4V, but biocompatibility was improved by replacing vanadium with niobium [66].

The Er^3+^:YAG laser may play a significant role in the process of decontaminating an infected implant surface [1,67,68]. However, the thermal rise associated with laser application can overheat tissues; thus, particular attention should be paid during the decontamination process. The composition of the peri-implant zone evokes diminished blood perfusion in the bone; hence, additional care around the implant is required. The studies in the animal model published by Eriksson et al. proved that a temperature increase in the bone by 10 °C for 1 min resulted in 10% cases of bone resorption in the 30-day follow-up [69]. Therefore, a temperature gradient below 10 °C should be recognized as innocuous to bone tissue. The maximal temperature varied across samples; the lowest was 27 °C in sample 4, while sample 8 had the highest, 79.2 °C. The authors also include, for comparative purposes, scans from a thermal imaging camera along with diagrams of the maximum temperature versus time and SEM images for selected samples (sample no. 1- Ti-V alloy, microscopic image unchanged, sample no. 8—Ti-Nb alloy, microscopic image unchanged, sample No. 10—Ti-Nb alloy, surface with damage features in the microscopic image). The results of Er^3+^:YAG laser irradiation at 80 mJ for both titanium grades (Ti-Nb and Ti-V) after 30 s of exposure showed that the temperature increased by less than 10 °C above the critical threshold. Our present results are in correspondence with the research of Monzavi et al., who stated the increase in titanium implants temperature of 4.30 °C for 1 min of Er^3+^:YAG laser exposure at a slightly lower mean power of 1W (100 mJ; 10Hz) [70]. Also, Kreisler et al. [71] obtained comparable results for Er^3+^:YAG laser radiation in the power range of 0.6–1.2 W. For all the samples irradiated in their study, the temperature remained at a stable high below 10 °C [72].

Current results indicate that both Ti6Al4V and Ti6Al7Nb alloys underwent successful laser decontamination without significant influence on their surface structure when the laser power was low (0.5 W). Under experimental conditions, laser power below 0.5 W allowed safe exposure without exceeding the 10 °C threshold; however, this value should not be considered an absolute limit for clinical applications. The maximum temperature during irradiation is strongly dependent on additional factors, such as the distance between the laser tip and the alloy surface and cooling conditions. Therefore, the power threshold may vary depending on these parameters. Furthermore, although reducing laser power minimizes thermal risks, too low a power can negatively impact surface decontamination efficiency. This compromise highlights the need for careful optimization of laser parameters in practical settings.

At higher laser power (3.2 W), SEM examinations revealed microscopic cracks on the surfaces of both alloys, which is a disadvantage for their mechanical properties, integrity, and resistance to bacterial accumulation. At a power of 9.6 W, the thermal rise exceeds the critical 10 °C, which is clinically unacceptable due to the risk of bone necrosis and tooth pulp injury [66,69]. It showed that the specimen surfaces changed with laser beam power. Increasing the lasing power was more effective than increasing the frequency of surface roughness [67], and it plays a determining role in surface topography. Rønold H.J. and Ellingsen J.E. et al. [69] suggested that the relationship between titanium surface topology and its mechanical properties, and that the first bone tissue bonds occur at surface roughness values of 0.5 to 1.5 μm. In our present study, the Er^3+^:YAG was applied onto the surface of the titanium samples by placing the tip at a distance of 10 mm (non-contact method), which decreases the risk of sample surface damage. Furthermore, the decontamination of the samples was under operator sight control; thus, the percentage of surface coverage by the laser beam was high. The percentage of the surface damage is laser power-dependent [66]. Matsuyama et al. [63] observed no morphological changes on the titanium surface under SEM evaluation at an energy below 50 mJ/pulse. In the current research, we also found no differences in the morphology of the titanium samples at the exact value of laser power; however, higher energies of 80 mJ and 160 mJ significantly influenced the surface of titanium implants, even when the laser pulse duration was 100 μm, which is two times less than in the study of Matsuyama et al. [63]. Moreover, no changes in the microstructure of the titanium surface during debridement with the Er^3+^:YAG laser were found in the study of Taniguchi et al. [72] at the power of 0.9 W (30 mJ, 30 Hz).

The findings of this study align with recent reports [49,73,74] emphasizing the need for safe laser parameters that achieve biofilm reduction without inducing thermal or morphological damage to titanium implant surfaces. Contemporary investigations similarly confirm that low-energy Er^3+^:YAG irradiation remains one of the most predictable approaches for preserving the microtopography essential for osseointegration.

Based on this research, samples made from Ti6Al4V and Ti6Al7Nb alloys can undergo Er^3+^:YAG laser exposure without significant temperature rise when the power does not exceed 0.5 W. However, the laser application to the surfaces of Ti6Al4V and Ti6Al7Nb alloys resulted in a temperature rise below the safety level of 100 °C at a power of 0.8 W (80 mJ, 10 Hz) after 30 s. Thermography results (see Figure 7) show a clear difference in cooling behavior between the large- and small-pore scaffolds, which can be directly attributed to their surface-to-volume (S/V) ratios. For example, when cooling, the small-pore structure (Figure 7E) exhibits strong spatial temperature fluctuations, with rapid transitions from hot to cold regions. This stark contrast reflects the rapid heat dissipation typical of high-S/V geometries, in which thermal energy is efficiently transferred to the surroundings. In contrast, the large-pore scaffold (see Figure 7B–D) is characterized by a much more uniform temperature field after cooling. Although this may appear more stable visually, it indicates slower, more uniform cooling, consistent with a lower S/V and a reduced total surface area available for heat transfer. These findings confirm that pore size—and the resulting surface-to-volume ratio—plays a dominant role in regulating the thermal degradation of the network structures during and after heating. Consequently, variations in pore dimensions or in material thermal conductivity (as observed in Ti6Al7Nb) would alter the local thermal history and the residual stress profile. Therefore, this study isolates the geometric influence by fixing the material parameters, with future comparative studies planned to address multi-material thermal behaviors.

Given the risk of bone resorption and titanium implant surface damage when using the Er^3+^:YAG laser, lower laser application parameters should be used in vivo. However, new randomized clinical trials are needed to confirm these findings in clinical practice.

## 5. Conclusions

The Er^3+^:YAG laser is a clinically acceptable method for decontaminating titanium alloys, provided that the device is carefully maintained during operation, particularly with respect to timing and exposure parameters, and that sufficient cooling is provided. Otherwise, if too high an energy is applied, it results in melting or cracks and microcracks, which, in addition to weakening the implant itself, provide a niche for colonization by bacteria that live in the mouth, increasing the risk of peri-implantitis.

The presented studies have shown that we successfully obtained implant blocks made out of Ti6Al4V and Ti6Al7Nb alloys, and that the structure of these scaffolds did not change significantly during the fabrication process—apart from possibly some minor β-phase, microstrains, or residual stresses, which were concluded from the XRD results. These implants can be successfully exposed to an Er^3+^:YAG laser with the following parameters: energy 50 mJ, power 0.5W, frequency 10 Hz, spot 0.9 mm, pulse width 100 µs, cooling 4 air; 6 water. However, it should be noted that the safe laser power threshold is not an absolute value, as it depends on factors such as the tip-to-surface distance and cooling conditions; furthermore, too low a power may impair decontamination efficiency, underscoring the need for further parameter optimization. Nevertheless, at higher laser energy values, we observed implant damage that was clearly visible in SEM images. Moreover, the EDS results indicate the formation of oxide phases at higher laser power. Regarding roughness, the surfaces of both Ti scaffolds did not change significantly at the lowest laser power. Based on thermograms, we found that the maximum temperature during laser irradiation was largely unaffected by the sample material, surface color, or exposure time, but was strongly dependent on the laser energy and cooling conditions.

## Figures and Tables

**Table 1:** Unit cell parameters (*a*, *c*), crystal cell volume (*V*), as well as the refine factor (*R_w_*) for the Ti^0^ powders and scaffolds, and the second phase TiO.

	Cell Parameters					Refine Factor
	Ti			TiO		
**Sample**	***a (Å)***	***c (Å)***	***V (Å*** **^3^** ***)***	***a (Å)***	***V (Å*** **^3^** ***)***	***R_w_ (%)***
**s. c.**	2.941 (1)	4.682 (2)	35.07 (3)	4.1912 (1)	73.62 (1)	–
**Volume fraction**		**100.0%**		**0.0%**		
**Ti6Al7Nb powder**	2.934 (0)	4.682 (3)	34.90 (7)	–	–	3.4
**Ti6Al4V powder**	2.931 (8)	4.670 (4)	34.76 (6)	–	–	3.2
**Ti6Al7Nb scaffold**	2.935 (8)	4.691 (6)	35.01 (9)	–	–	3.3
**Ti6Al4V scaffold**	2.926 (6)	4.661 (0)	34.57 (3)	–	–	3.8
**Ti6Al7Nb scaffold (50 mJ)**	2.937 (6)	4.692 (1)	35.06 (6)	–	–	3.6
**Volume fraction**		**73.1%**		**26.9%**		
**Ti6Al7Nb scaffold (320 mJ)**	2.940 (6)	4.703 (2)	35.22 (0)	4.1876 (8)	73.43 (8)	3.4
**Volume fraction**		**100.0%**		**0.0%**		
**Ti6Al4V scaffold (50 mJ)**	2.928 (9)	4.666 (9)	34.67 (1)	–	–	3.5
**Ti6Al4V scaffold (320 mJ)**	2.932 (3)	4.672 (7)	34.79 (5)	–	–	3.8

s. c.—single crystal reference data, Ti-ICSD-191187, TiO-ICSD-38755.

**Table 2:** Chemical composition (wt.%) of Ti6Al7Nb scaffold sample after laser exposure determined by EDS technique.

*E* (mJ)	Chemical Composition (wt. %) ^(^*^)^							
	20 s				30 s			
	Ti	Al	Nb	O	Ti	Al	Nb	O
50	87.1	6.2	6.3	0.4	87.0	6.0	6.1	0.9
80	86.6	6.0	6.1	1.4	84.1	6.3	5.7	3.9
160	75.1	4.6	4.6	15.7	76.5	5.0	4.9	13.6
320	74.1	3.8	3.6	18.5	74.0	3.4	3.6	18.9

(*) The relative errors of the EDS method are less than 2%, 4% and 50% for main (above 20 at. %), major (20–5. at. %), and trace (1–0.1) elements, respectively.

**Table 3:** Samples “b” (bright) and “m” (matte) differed in surface roughness (emissivity factor). “m” and “b” consider the surface finish of the material: “m” indicates a matte surface, while “b” indicates a bright surface. The emissivity factor of the bright surface is *ε_b_* = 0.27, and the emissivity factor of the matte surface is *ε_m_* = 0.75. *Me*—median, *Q*_1_—lower quartile, *Q*_3_—upper quartile.

Material	*t*_exp_. (s)	*E* (mJ)	*f* (Hz)	*P* (W)	Water Cooling	*T_max_* (°C)	
						*Max*	*Me* (*Q*_1_; *Q*_3_)
Ti6Al4V “m”	20	160	10	1.6	yes	28.0	25.7 (24.9; 25.8)
Ti6Al4V “m”	30	160	10	1.6	yes	29.4	25.5 (24.9; 26.5)
Ti6Al4V “b”	30	160	10	1.6	yes	33.1	25.3 (24.4; 27.0)
Ti6Al4V “b”	30	160	10	1.6	yes	38.2	25.3 (24.3; 25.8)
Ti6Al4V “b”	30	80	10	0.8	yes	27.0	24.3 (24.2; 25.0)
Ti6Al4V “m”	30	80	10	0.8	yes	26.5	24.4 (24.2; 24.8)
Ti6Al4V “b”	30	320	30	9.6	yes	46.2	29.5 (27.1; 31.6)
Ti6Al4V “m”	30	320	30	9.6	yes	42.4	29.0 (27.4; 31.5)
Ti6Al7Nb	30	320	30	1.6	no	79.2	36.4 (31.3; 46.3)
Ti6Al7Nb	30	320	30	1.6	yes	32.7	25.2 (24.5; 26.7)
Ti6Al7Nb	30	80	10	0.8	yes	27.5	25.0 (24.5; 25.9)

## Data Availability

The original contributions presented in this study are included in the article/Supplementary Material. Further inquiries can be directed to the corresponding authors.

## Supplementary Materials

The following supporting information can be downloaded at: https://www.mdpi.com/article/10.3390/ma19040775/s1, Figure S1: Representative results of Ti^0^ scaffold laser exposure (320 mJ), Rietveld analysis (red–fitted diffraction; blue—differential pattern, column—reference phase peak positions); Figure S2: Different variants of laser irradiation on Ti-cubic scaffolds (Ti6Al4Nb and Ti6Al4V as indicated in Figure 1. Variant I: 80 mJ, 0.8 W, 10 Hz, 0.9 mm, 100 µs, cooling 4 air; 6 water; Variant II: 160 mJ, 1.6 W, 10 Hz, 0.9 mm, 100 µs, cooling 4 air; 6 water; Variant III: 320 mJ, 9.6 W, 30 Hz, 0.9 mm, 100 µs, cooling 4 air; 6 water. The red marks indicate the locations of laser exposure: one red mark on the scaffold wall means the laser was used for 20 s, and two red marks on the scaffold wall mean the laser was exposed for 30 s. The last Variant IV is defined by the following laser parameters: 50 mJ, 0.5W, 10 Hz, 0.9 mm, 100 µs, cooling: 4 air; 6 water; 30 s. of laser exposure; Figure S3: SEM images of Ti6Al7Nb scaffold after 20 s of Er: YAG laser exposure (80 mJ/0.8 W; 160 mJ/1.6 W; 320 mJ/3.2 W) compared to areas without laser exposure; Figure S4: SEM images of Ti6Al7Nb scaffold after 30 s of Er: YAG laser exposure (80 mJ/0.8 W; 160 mJ/1.6 W; 320 mJ/3.2 W) compared to areas without laser exposure; Figure S5: SEM images of Ti6Al4V scaffold after 20 s of Er: YAG laser exposure (80 mJ/0.8 W; 160 mJ/1.6 W; 320 mJ/3.2 W) compared to areas without laser exposure; Figure S6: SEM images of Ti6Al4V scaffold after 30 s of Er: YAG laser exposure (80 mJ/0.8 W; 160 mJ/1.6 W; 320 mJ/3.2 W) compared to areas without laser exposure. A, B, and C show Area A, Area B, and Area C, respectively, as indicated in a legend panel; Table S1: Chemical composition (wt.%) of Ti6Al4V scaffold sample after laser exposure determined by EDS technique.
