# Supplementary material for: The Effect of the Er3+:YAG Laser Decontamination Process on the Surface of Titanium Alloys, Depending on the Exposure Parameters
Source: Materials (Basel). 2026 Feb 16;19(4):775. doi: 10.3390/ma19040775 (PMC12942487; doi:10.3390/ma19040775)
Supplement: Supplementary file 1 [file materials-19-00775-s001.zip › materials-4125545-supplementary.pdf]

materials

MDPI

Supplementary Information

Article

The Effect of the Er^{3+}:YAG Laser Decontamination Process on the Surface of Titanium Alloys, Depending on the Exposure Parameters

Paulina Sobierajska [1,*], Maciej Dobrzynski [2], Kinga Grzech-Lesniak [3,4], Kinga Sekula [5], Damian Szymanski [1], Wojciech Zakrzewski [6], Krzysztof D. Dudek [7], Jacek Matys [8], Maria Szymonowicz [6], Katarzyna Wiglusz [9], Dawid Keszycki [5] and Rafal J. Wiglusz [2,10,*]

[1] Institute of Low Temperature and Structure Research, Polish Academy of Sciences, Okolna 2, 50-422 Wroclaw, Poland; d.szymanski@intibs.pl
[2] Department of Pediatric Dentistry and Preclinical Dentistry, Wroclaw Medical University, 50-425 Wroclaw, Poland; maciej.dobrzynski@umw.edu.pl
[3] Department of Integrated Dentistry, Faculty of Medicine and Dentistry, Wroclaw Medical University, Krakowska 26, 50-425 Wroclaw, Poland; kinga.grzech-lesniak@umw.edu.pl or kgl@periocare.pl
[4] Department of Periodontics, School of Dentistry, Virginia Commonwealth University, Richmond, VA, 23298-0566, USA
[5] Department of Advanced Manufacturing Technologies, Faculty of Mechanical Engineering, Wroclaw University of Science and Technology, Lukasiewicza 5, 50-371 Wroclaw, Poland; kinga.sekula@pwr.edu.pl (K.S.); dawid.keszycki@pwr.edu.pl (D.K.)
[6] Pre-Clinical Research Centre, Wroclaw Medical University, Bujwida 44, 50-368 Wroclaw, Poland; wojciech.zakrzewski1992@gmail.com (W.Z.); maria.szymonowicz@umw.edu.pl (M.S.)
[7] Department of Logistics and Transport Systems, Faculty of Mechanical Engineering, Wroclaw University of Science and Technology, 50-371 Wroclaw, Poland; krzysztof.dudek@pwr.edu.pl
[8] Department of Dental Surgery, Faculty of Medicine and Dentistry, Wroclaw Medical University, Krakowska 26, 50-425 Wroclaw, Poland; jacek.matys@umw.edu.pl
[9] Department of Basic Chemical Sciences, Faculty of Pharmacy, Wroclaw Medical University, Borowska 211 A, 50-566 Wroclaw, Poland; katarzyna.wiglusz@umw.edu.pl
[10] Meinig School of Biomedical Engineering, College of Engineering, Cornell University, Ithaca, NY 14853-1801, USA
* Correspondence: p.sobierajska@intibs.pl (P.S.); r.wiglusz@intibs.pl or rjw283@cornell.edu (R.J.W.); Tel.: +48-071-395-42-74 (P.S.); +48-071-395-41-59 (R.J.W.); Fax: +48-071-344-10-29 (R.J.W.)

Abstract

The dynamic development of laser therapy in dentistry is associated, among other factors, with the bactericidal effect of the energy emitted by laser devices. Therefore, they are also helpful for decontamination. They are increasingly used in the treatment of peri-implantitis, a bacterial inflammation of peri-implant tissues that is the most severe late complication of implantation and a potential cause of implant loss. Therefore, this study aimed to assess the safety of laser decontamination of the implant surface with respect to its effect on the integrity of the implant structure. In the present study, blocks of the titanium alloys Ti-6Al4V and Ti6Al7Nb were fabricated using electron-beam powder bed fusion and laser powder bed fusion, respectively. These alloys, commonly used in implantology, here in the form of Ti block scaffolds, have been exposed to an Er^{3+}:YAG laser under various parameters (energy range of 50-320 mJ, exposure times of 20 or 30 sec.) and their effects have been further observed. To determine the changes induced by the laser, the following techniques were used: X-ray diffraction (XRD), Rietveld refinement method, scanning electron microscopy (SEM) with EDS (Energy-dispersive X-ray Spectroscopy), and thermography.

 https://doi.org/10.3390/xxxxx

The results show that the proposed Ti6Al4V and Ti6Al7Nb scaffolds can be exposed to an Er^{3+}:YAG laser without damage when the power is limited to 0.5 W.

Keywords: decontamination, Er^{3+}:YAG, laser, peri-implantitis, Ti6Al4V, and Ti6Al7Nb alloys

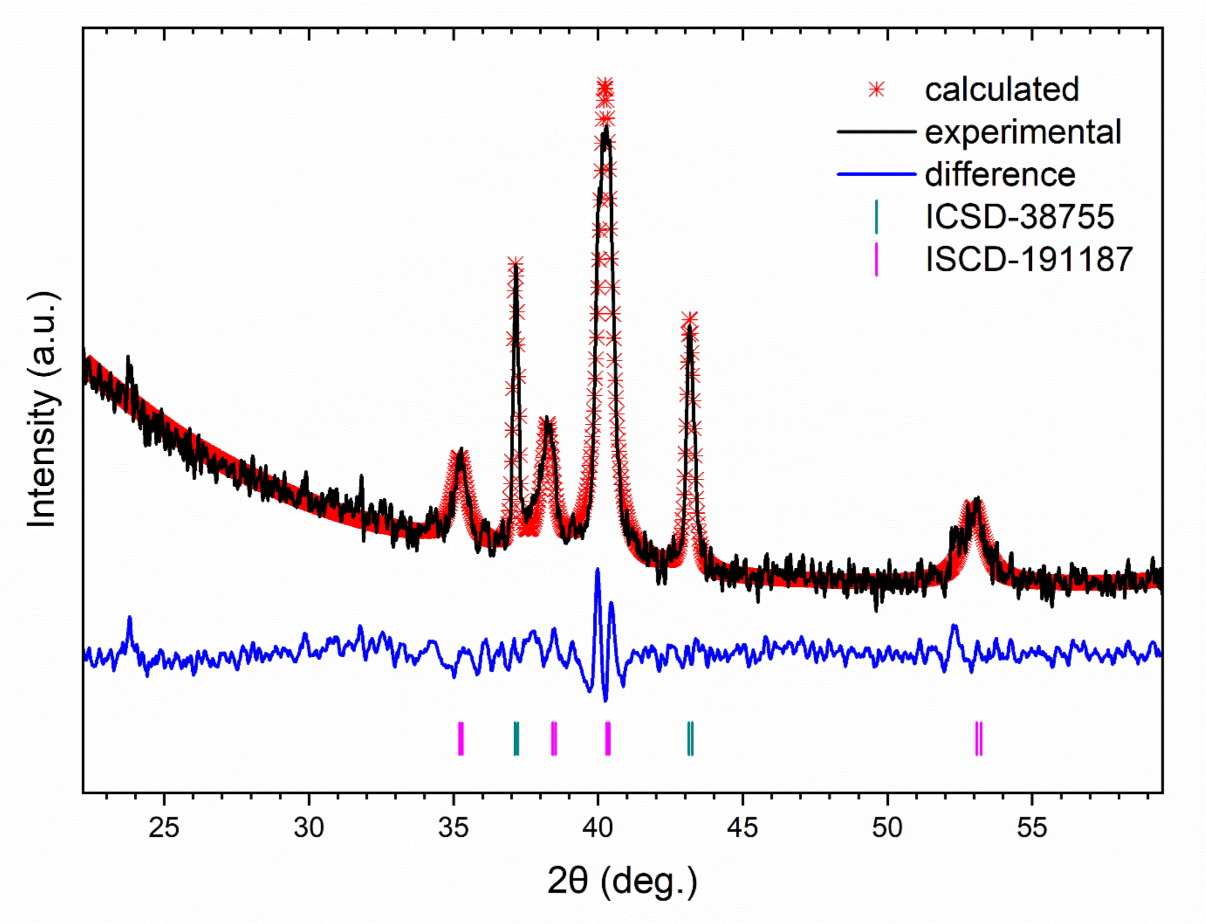

Figure S1. Representative results of Ti^0 scaffold laser exposure (320mJ), Rietveld analysis (red–fitted diffraction; blue – differential pattern, column – reference phase peak positions).

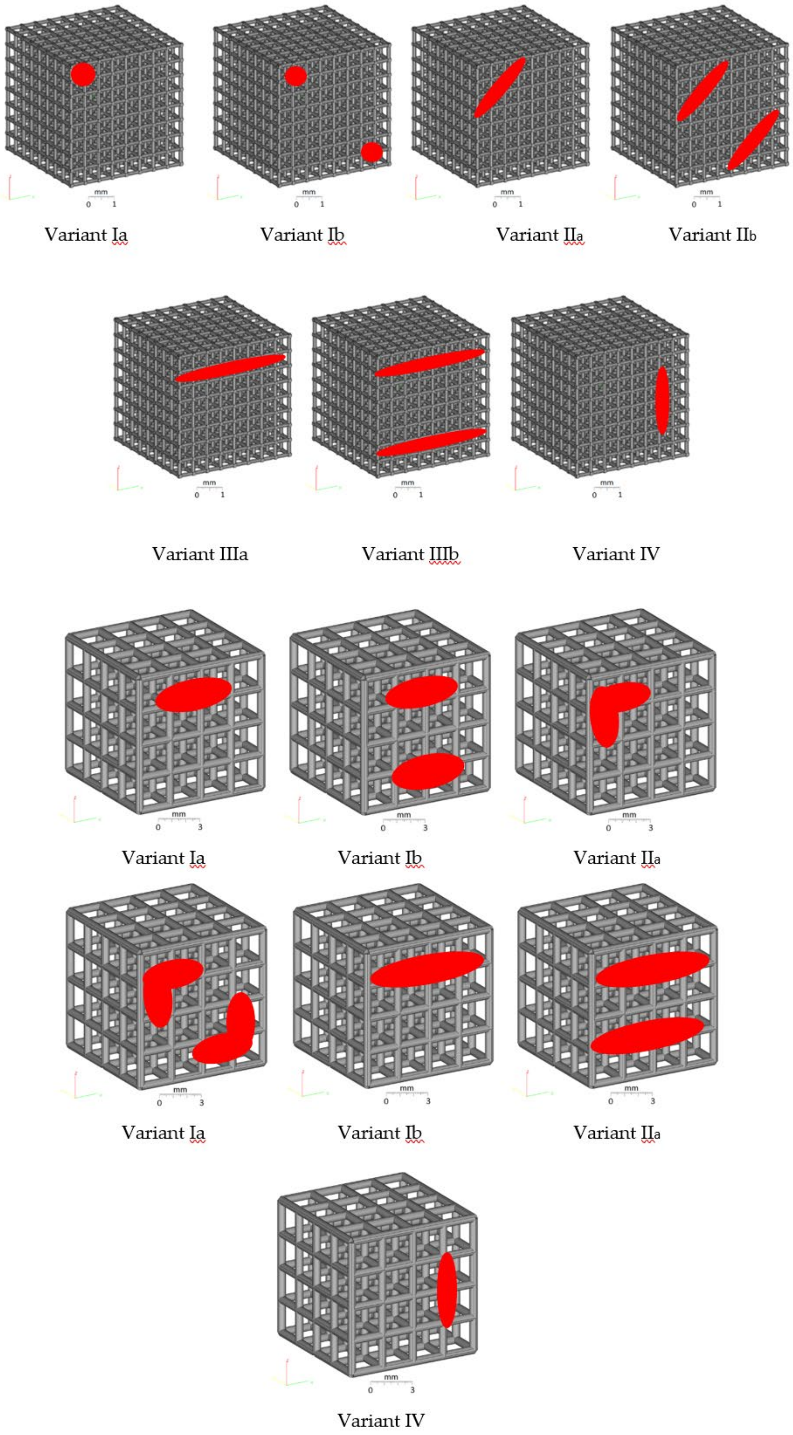

Figure S2. Different variants of laser irradiation on Ti-cubic scaffolds (Ti6Al4Nb and Ti6Al4V as indicated in Figure 1. Variant I: 80 mJ, 0.8 W, 10 Hz, 0.9 mm, 100 µs, cooling 4 air; 6 water; Variant II: 160 mJ, 1.6 W, 10 Hz, 0.9 mm, 100 µs, cooling 4 air; 6 water; Variant III: 320 mJ, 9.6 W, 30 Hz, 0.9 mm, 100 µs, cooling 4 air; 6 water. The red marks indicate the locations of laser exposure: one red mark on the scaffold wall means the laser was used for 20 seconds, and two red marks on the scaffold wall mean the laser was exposed for 30 seconds. The last Variant IV is defined by the following laser parameters: 50 mJ, 0.5W, 10 Hz, 0.9 mm, 100 µs, cooling: 4 air; 6 water; 30 sec. of laser exposure.

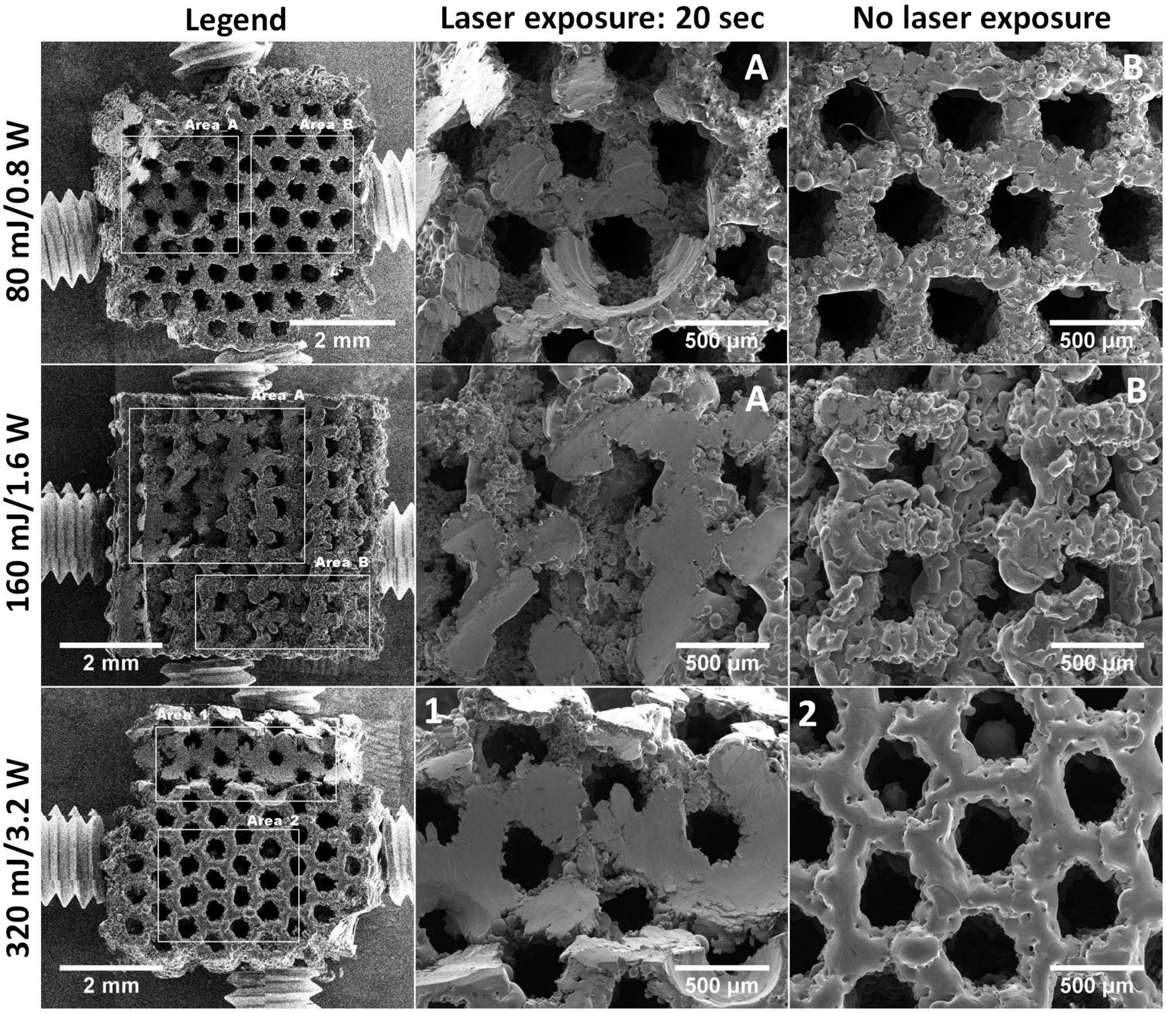

Figure S3. SEM images of Ti6Al7Nb scaffold after 20 seconds of Er: YAG laser exposure (80mJ/ 0.8W; 160mJ/1.6W; 320 mJ/3.2W) compared to areas without laser exposure.

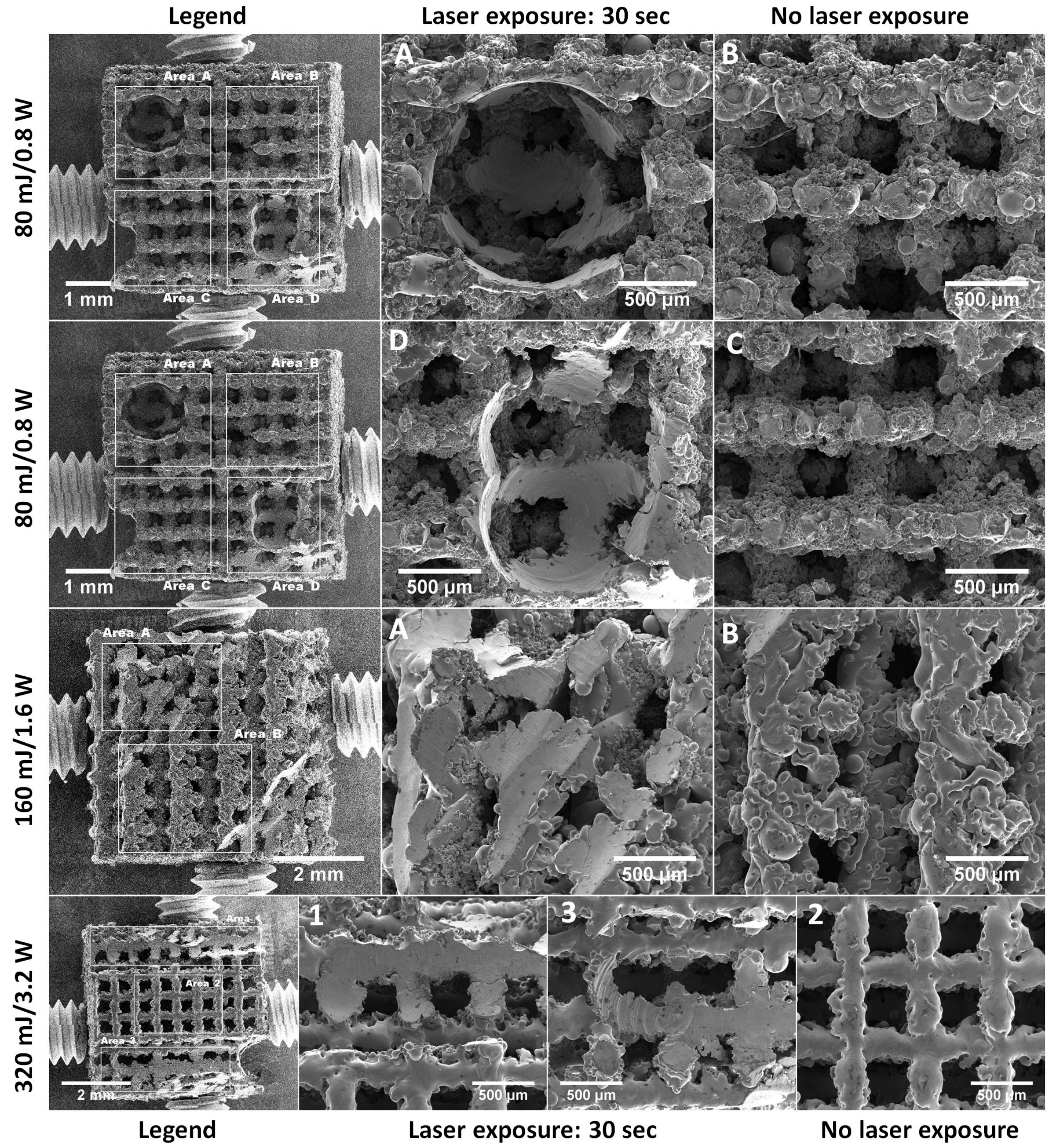

Figure S4. SEM images of Ti6Al7Nb scaffold after 30 seconds of Er: YAG laser exposure (80mJ/ 0.8W; 160mJ/1.6W; 320 mJ/3.2W) compared to areas without laser exposure.

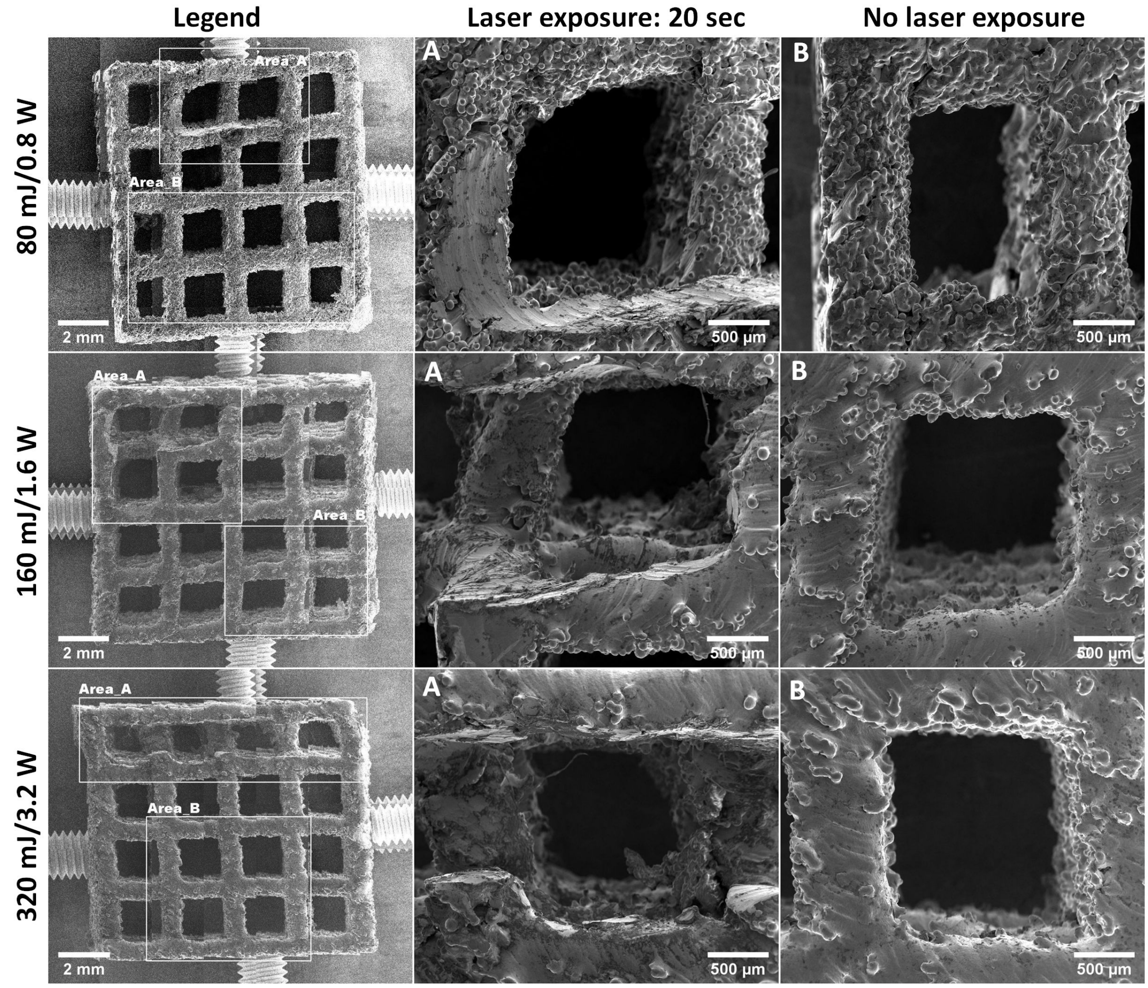

Figure S5. SEM images of Ti6Al4V scaffold after 20 seconds of Er: YAG laser exposure (80mJ/ 0.8W; 160mJ/1.6W; 320 mJ/3.2W) compared to areas without laser exposure.

https://doi.org/10.3390/xxxxx

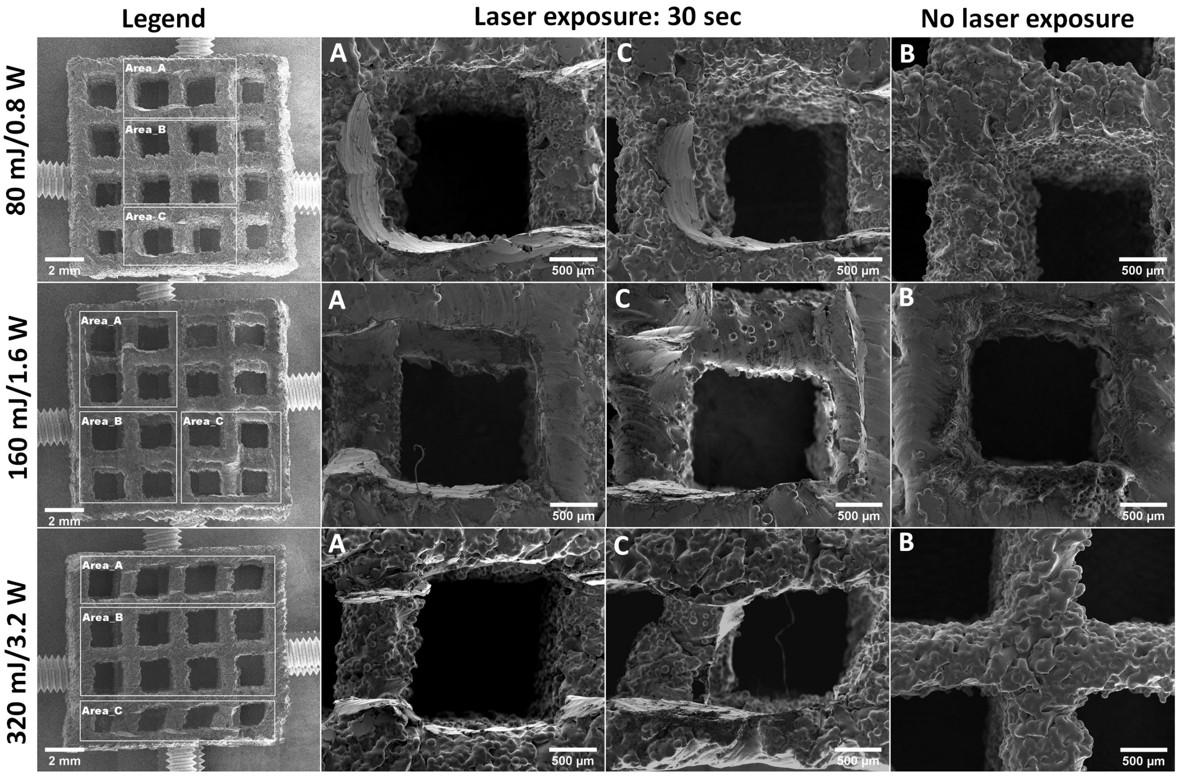

Figure S6. SEM images of Ti6Al4V scaffold after 30 seconds of Er: YAG laser exposure (80mJ/ 0.8W; 160mJ/1.6W; 320 mJ/3.2W) compared to areas without laser exposure. A, B, and C show Area A, Area B, and Area C, respectively, as indicated in a legend panel.

Table S1. Chemical composition (wt.%) of Ti6Al4V scaffold sample after laser exposure determined by EDS technique.

Power (mJ)	Chemical composition (wt. %)(*)							
	20 s				30 s			
	Ti	Al	V	O	Ti	Al	V	O
50	86.3	7.9	3.4	2.3	87.3	7.7	3.3	1.6
80	86.8	7.6	3.4	2.2	85.4	8.0	3.1	3.4
160	83.8	7.5	3.0	5.6	81.9	6.8	3.2	8.2
320	85.9	6.7	2.9	4.5	81.6	6.1	2.9	9.4

(*) The relative errors of the EDS method are less than 2 %, 4 %, and 50% for main (above 20 at.% %), major (20 - 5 at.% %), and trace (1 - 0.1) elements, respectively
